# Tinea. Sulph. Acid

**Published:** 1854-12

**Authors:** 


Tinea. Sulphurous Acid.—M. Verhaeghe relates three cases of tinea treated by the local application of sulphurous acid, after the manner of Jenner. In two cases a rapid cure was effected; in the third case there was temporary amelioration, but the disease subsequently returned.

M. Bagin has published a memoir on tinea, to show the success of his treatment. This consists of the most careful epilation, and the destruction of the parasitic plant by a solution of the bichloride of mercury, or of the acetate of copper. The details of the treatment are as follows: The hair is first cut close; sulphur lotions and cataplasms to attack the crusts are applied; the solution of corrosive sublimate is then applied so as to kill all the plant on the surface. Then either the hairs are pulled out with pincers, or if the disease be recent and the epilation difficult, the oil of cade, or an alkaline pomade of lime and soda, is rubbed on. When a certain space has been cleared of hair, it is washed with soap and warm water to get rid of fat, and then the parasiticide solution (1 part of corrosive sublimate or of acetate of copper to 100 of water) is immediately applied.—*Revue Med. Chir. de Paris.*

